# Inhibition of TMEM16A Expression Suppresses Growth and Invasion in Human Colorectal Cancer Cells

**DOI:** 10.1371/journal.pone.0115443

**Published:** 2014-12-26

**Authors:** Yujie Sui, Meiyan Sun, Fei Wu, Longfei Yang, Weihua Di, Guizhen Zhang, Lili Zhong, Zhiming Ma, Jinhao Zheng, Xuedong Fang, Tonghui Ma

**Affiliations:** 1 Key Laboratory for Molecular and Chemical Genetics of Critical Human Diseases of Jilin Province, Jilin University Bethune Second Hospital, Changchun, P. R. China; 2 Department of Gynecology and Obstetrics, Jilin University Bethune Second Hospital, Changchun, P. R. China; 3 Department of General Surgery, Jilin University Bethune Second Hospital, Changchun, P. R. China; 4 Department of General Surgery, China-Japan Friendship Hospital of Jilin University, Changchun, P. R. China; 5 College of Basic Medical Sciences, Dalian Medical University, Dalian, P. R. China; Institute of Molecular and Cell Biology, Biopolis, United States of America

## Abstract

Metastasis leads to poor prognosis in colorectal cancer patients, and there is a growing need for new therapeutic targets. TMEM16A (ANO1, DOG1 or TAOS2) has recently been identified as a calcium-activated chloride channel (CaCC) and is reported to be overexpressed in several malignancies; however, its expression and function in colorectal cancer (CRC) remains unclear. In this study, we found expression of TMEM16A mRNA and protein in high-metastatic-potential SW620, HCT116 and LS174T cells, but not in primary HCT8 and SW480 cells, using RT-PCR, western blotting and immunofluorescence labeling. Patch-clamp recordings detected CaCC currents regulated by intracellular Ca^2+^ and voltage in SW620 cells. Knockdown of TMEM16A by short hairpin RNAs (shRNA) resulted in the suppression of growth, migration and invasion of SW620 cells as detected by MTT, wound-healing and transwell assays. Mechanistically, TMEM16A depletion was accompanied by the dysregulation of phospho-MEK, phospho-ERK1/2 and cyclin D1 expression. Flow cytometry analysis showed that SW620 cells were inhibited from the G1 to S phase of the cell cycle in the TMEM16A shRNA group compared with the control group. In conclusion, our results indicate that TMEM16A CaCC is involved in growth, migration and invasion of metastatic CRC cells and provide evidence for TMEM16A as a potential drug target for treating metastatic colorectal carcinoma.

## Introduction

Colorectal cancer (CRC) is the third most common malignancy worldwide [1], [2], and metastasis is a crucial factor for the poor prognosis of CRC patients [3]. Alterations of multiple gene, such as activation of oncogenes and inactivation of tumor suppressor genes, are involved in the progression of normal colonic epithelium into adenoma and into malignant adenocarcinoma [4], [5] However, there is limited information about the molecular changes that confer to the colorectal cancer metastasis [6], [7]. Therefore, it is necessary to identify metastasis-related genes and their molecular pathways, which may provide new targets for the treatment of metastatic CRC.

The chromosomal band 11q13 amplicon is one of the most frequently amplified regions in human malignancies, such as head and neck squamous cell carcinoma (HNSCC) and breast, bladder and esophageal cancer [8]. The analysis of the differential expression of genes located in this region led to the identification of *TMEM16A*, which is also called *ANO1* (anoctamin-1), *DOG1* (discovered on gastrointestinal stromal tumors protein 1), *ORAOV2* (oral cancer overexpressed 2) and *TAOS2* (tumor-amplified and overexpressed sequence 2) [9], [10], [11], [12], [13]. TMEM16A is composed of 26 exons and contains eight transmembrane segaments with the N- and C-termini faced the cytoplasm and a reentrant loop located between TM5 and TM6 possibly forming the pore region [14].

TMEM16A has recently been shown to be a calcium-activated chloride channel [14], [15], [16] and is widely expressed in various tissues, including secretory epithelia, smooth muscle, sensory neurons and other tissues [17], [18]. TMEM16A plays many important physiological roles in the control of epithelial fluid transport, vascular smooth muscle contraction, saliva production and gastrointestinal tract motility [19], [20], [21], [22]. Dysregulation of TMEM16A causes human diseases, including cystic fibrosis, hypertension, pulmonary diseases and diarrhea [23], [24], [25]; knockout of TMEM16A is embryonically lethal because of tracheomalacia [26].

The expression of TMEM16A is up-regulated in several cancers, including HNSCC and esophageal, breast and prostate cancer. Its overexpression is also correlated with the development of distant metastasis and poor prognosis of cancer patients with HNSCC [27], [28], [29]. Recently, TMEM16A has been found to promote HNSCC tumorigenesis and invasion via activating the mitogen-activated protein kinase (MAPK) signaling pathway. In addition, TMEM16A has been reported to contribute to cancer progression by inducing the activation of epithelial growth factor receptor (EGFR) and calmodulin-dependent protein kinase II (CAMK II) and subsequently activating AKT and MAPK signaling in breast cancer and HNSCC [30], [31]. Although TMEM16A is ubiquitously expressed in gastrointestinal stromal tumors [32], its role in CRC metastasis is little investigated.

In the present study, we first demonstrated the expression of TMEM16A calcium-activated chloride channels (CaCCs) in different metastatics potential colorectal cancer cell lines. We further investigated role of TMEM16A in SW620 cells metastasis and its possible molecular mechanism by using short hairpin RNAs in vitro.

## Materials and Methods

### Cell culture

The human colorectal carcinoma cell lines HCT8, SW480, SW620, HCT116 and LS174T cells were obtained from the American Type Culture Collection (ATCC). SW480 and SW620 were cultured in L15 Medium (sigma, USA). HCT8 and HCT116 were grown in RPMI medium 1640 (sigma, USA). LS174T cells were cultured in Dulbecco's modified Eagle's medium (Sigma, USA). Fisher rat thyroid (FRT) cells and FRT cells transfected stably with human TMEM16A were obtained from Alan Verkman (University of California, San Francisco, CA, USA) [33] and were cultured in Coon's modified F12 medium. All media was supplemented with 10% fetal bovine serum, 100 U/ml penicillin and 100 µg/ml streptomycin. Cells were incubated at 37°C in a humidified atmosphere of 5% CO_2_ and 95% air.

### RNA extraction and RT-PCR

Total RNA was extracted from cells using TRIzol reagent (Invitrogen, Carlsbad, CA, USA) according to the manufacturer's introductions. The concentration, purity and integrity of the RNA were measured using a NanoDrop2000 spectrophotometer (Thermo Scientific). Five hundred nanograms of total RNA was reverse transcribed to cDNA using the reverse transcriptase with the PrimeScriptTM RT reagent kit (TaKaRa). The cDNA product was used as a template for PCR amplification in a total volume of 30 µl, and the PCR conditions were as follows: 94°C for 5 min, followed by 30 cycles of 94°C for 30 s; 60°C for 30 s; and 72°C for 60 s. For TMEM16A cDNA amplification, the following primers were used: sense primer, 5′ –AACGGGA CCATGCACGGCTT-3′; antisense primer, 5′ -TGTTGTGGTGGTTGCACGGC-3′. The PCR products (424 bp) were analyzed by agarose gel electrophoresis, and β-actin served as an endogenous control to normalize the expression data.

### Western blotting

Cells were washed twice with ice-cold phosphate-buffered saline (PBS) and dissolved in lysis buffer (150 mM NaCl, 20 mM Tris, 5 mM EDTA, pH 7.5, 1% Triton X-100 and supplemented with 1 mM PMSF). After protein quantification, samples containing 100 µg proteins were denatured and electrophoresed using 10% SDS-PAGE and then transferred onto PVDF membranes. After blocking with 5% (w/v) nonfat milk and washing with Tris-buffered saline–Tween solution (TBST), the membranes were incubated overnight at 4°C with primary anti-TMEM16A polyclonal antibody (Abcam, ab53212; 1∶100) and mouse anti-β-actin antibody (Cell Signaling; 1∶500), respectively. All other primary antibodies were purchased from Cell Signaling Technology. After washing, the blots were incubated with secondary antibody conjugated to horseradish peroxidase (Sigma; 1∶5,000) for 1 h at room temperature and visualized using an enhanced chemiluminescence kit (Amersham, Little Chalfont, UK) on X-ray film (Millipore Corporation, Billerica, USA).

### Immunofluorescence analysis

Immunofluorescent staining was conducted to localize the expression of TMEM16A. Colorectal cancer cells were cultured overnight on preferred glass coverslips (Fisher, USA) and fixed for 15 min in 4% (w/v) paraformaldehyde (PFA). The cells were then rinsed 3 times with PBS for 5 min, permeabilized with 0.5% Triton X-100/PBS for 20 min and then incubated for 1 h in 3% BSA/PBS blocking solution. To detect TMEM16A, cells were incubated at 37°C for 3 h with mouse anti-human DOG1 monoclonal antibody (zhongshanjinqiao, China; 1∶50), followed by exposure to a Cy3-conjugated anti-mouse IgG secondary antibody (Sigma; 1∶1,000) at 37°C for 1 h. To visualize nuclei, cells were stained with DAPI (Sigma, St Louis, MO, USA; 40,6- diamidino-2-phenylindole), and the coverslips were mounted on slides with antifade solution (Vector Laboratories, Inc. Burlingame, CA). Fluorescent images were examined and photographed under a confocal microscope (Olympus, Tokyo, Japan).

### Patch clamp recordings

Human colorectal cancer cells were seeded on glass coverslips, and patch clamp recordings were performed using an EPC10 amplifier (HEKA, Lambrecht/Pfalz, Germany) in combination with Patchmaster (HEKA) at room temperature. The pipettes were prepared from borosilicate capillary glass using a micropipette puller (PC-10, NARISHIGE, Japan) and then fire-polished with a microforge (MF-900, NARISHIGE, Japan). The resistance of the pipettes in the bath solution was 2–5 MΩ. Cells were perfused with a bath solution containing: 145 mM NaCl, 5 mM KCl, 2 mM MgCl_2_, 1 mM CaCl_2_, 5 mM glucose, 5 mM HEPES and 20 mM sucrose (pH 7.4 with NaOH). For the whole-cell configuration, the zero Ca^2+^ intracellular solution contained the following: 146 mM N-methyl-D-glucamine chloride (NMDG-Cl), 2 mM MgCl_2_, 5 mM EGTA and 8 mM HEPES (pH 7.4 with NMDG). The high-Ca^2+^ pipette solution contained 5 mM Ca^2+^-EGTA instead of EGTA (free Ca^2+^ approximately 25 µM). Different free [Ca^2+^] solutions were made by mixing the zero-Ca^2+^ and high-Ca^2+^ solutions. The cell was clamped from the holding potential (0 mV) to voltages between −100 to +100 mV at 20 mV increments. For the inside-out configuration, the pipette solution contained: 140 mM NMDG-Cl, 2 mM MgCl_2_, 5 mM CaCl_2_ and 10 mM HEPES (pH 7.4 with NMDG); and the perfusion solution contained: 150 mM NMDG-Cl, 2 mM MgCl_2_, 10 mM EGTA, 8 mM Tris and 10 mM HEPES (pH 7.4 with NMDG). After the seal resistance reached >40 GΩ, the membrane was excised, and the membrane potential was held at −50 mV. The data were filtered at 100 Hz with an eight-pole Bessel filter (LPF-8, Warner Instruments, LLC, Hamden, CT, USA), and digitized using a computer at a sampling rate of 500 Hz. Niflumic acid (NFA) was purchased from Sigma-Aldrich. All inside-out patch experiments were performed using a fast solution exchange perfusion system (SF-77B, Warner Instruments). The dead time of solution change was 30 ms, and data analysis was performed for recordings containing whole cells in a single channel using the Igor software as described previously [34].

### RNA interference

TMEM16A/ANO1 short hairpin RNA (shRNA) and negative-control shRNA plasmid were constructed by GeneChem Co., Ltd. (Shanghai, China). The site for siRNA targeting of the human TMEM16A/ANO1 gene (GenBank NM_018043) was as follows: #1, CGTGTACAAAGGCCAAGTA (1077–1095 nt); and #2, CGAAGAAGA TGTACCACAT (837–855 nt). RNA interference was conducted according to the manufacturer's instructions.

### Cell proliferation assay

Cell proliferation rates were measured using the MTT assay. Briefly, 5000 cells were seeded in each 96-well plate for 24 h, transfected with TMEM16A shRNA or scrambled shRNA and further incubated for 1, 2, 3 and 4 d, respectively. MTT reagent (10 µl; 500 µg/mL) was added to each well, and the cells were further incubated for 4 h. Subsequently, 150 µl DMSO was added to dissolve the formazan crystals. The number of viable cells was quantified using the absorbance measured at 570 nm with a microplate reader (Thermo Scientific, USA).

### *In vitro* wound healing

Cell mobility was assessed by a scratch wound assay in vitro. Cells were seeded in a 6-well plate until confluent, scraped with a sterile, 200-µl tip and washed twice with PBS. After incubation with L15 medium containing 1% fetal bovine serum the cells were photographed at 0 h, 24 h, 48 h or 72 h under an inverted microscope (Olympus, Tokyo, Japan) at a magnification of 100×. These experiments were carried out in triplicate. The distances between the wound edges were measured with a graduated ruler, and relative scratch breadth was determined by a ratio of average breadth in treatment cells versus the average breadth in control cells.

### Cell migration and invasion assays

*In vitro* cancer cell migration and invasion activities were evaluated in transwells, as described previously, with modifications [23]. SW620 cells were cultured in L15 medium with 10% FBS until confluence in 6-well plates, transfected with the TMEM16A shRNA or scrambled shRNA for 24 h, and then trypsinized, washed and counted. For cell migration, 1×10^5^ cells in 200 µl of medium with 1% FBS were seeded in the upper transwell insert chamber containing a polycarbonate filter (6.5-mm diameter, 8-µm pores; Corning Costar, Corning, NY, USA). L15 medium (600 µl) with 10% FBS (chemoattractant) was added to the lower chamber, and the plates were incubated for 72 h at 37°C in 5% CO_2_. For cell invasion, the transwells were coated with Matrigel. The cells that did not migrate were removed from the top of the transwell filters by scraping. The penetrated cells were fixed with paraformaldehyde, stained with Coomassie blue and counted under an inverted microscope (100× magnification). The cell number represented migration activity.

### Cell-cycle analysis

After transfecting the cells with TMEM16A or scrambled shRNAs for 3 d, the cells were detached using trypsin, resuspended in growth medium and counted. Then, 1×10^6^ cells were washed with PBS and fixed overnight with 70% (vol/vol) ethanol at 4°C. After washing twice with PBS, the cells were stained with a solution containing 50 µg/mL of PI and 100 µg/mL RNase A for 30 min in the dark at room temperature. The stained cells were analyzed by flow cytometry (Beckman Coulter, Epics XL).

### Statistical analysis

The statistical analysis was performed using SPSS statistics (version 17.0) with a two-tailed Student's *t*-test. *P*<0.05 was considered statistically significant. The data are expressed as the mean ± *SD*.

## Results

### Expression of TMEM16A in CRC cell lines

**Figure 1:**
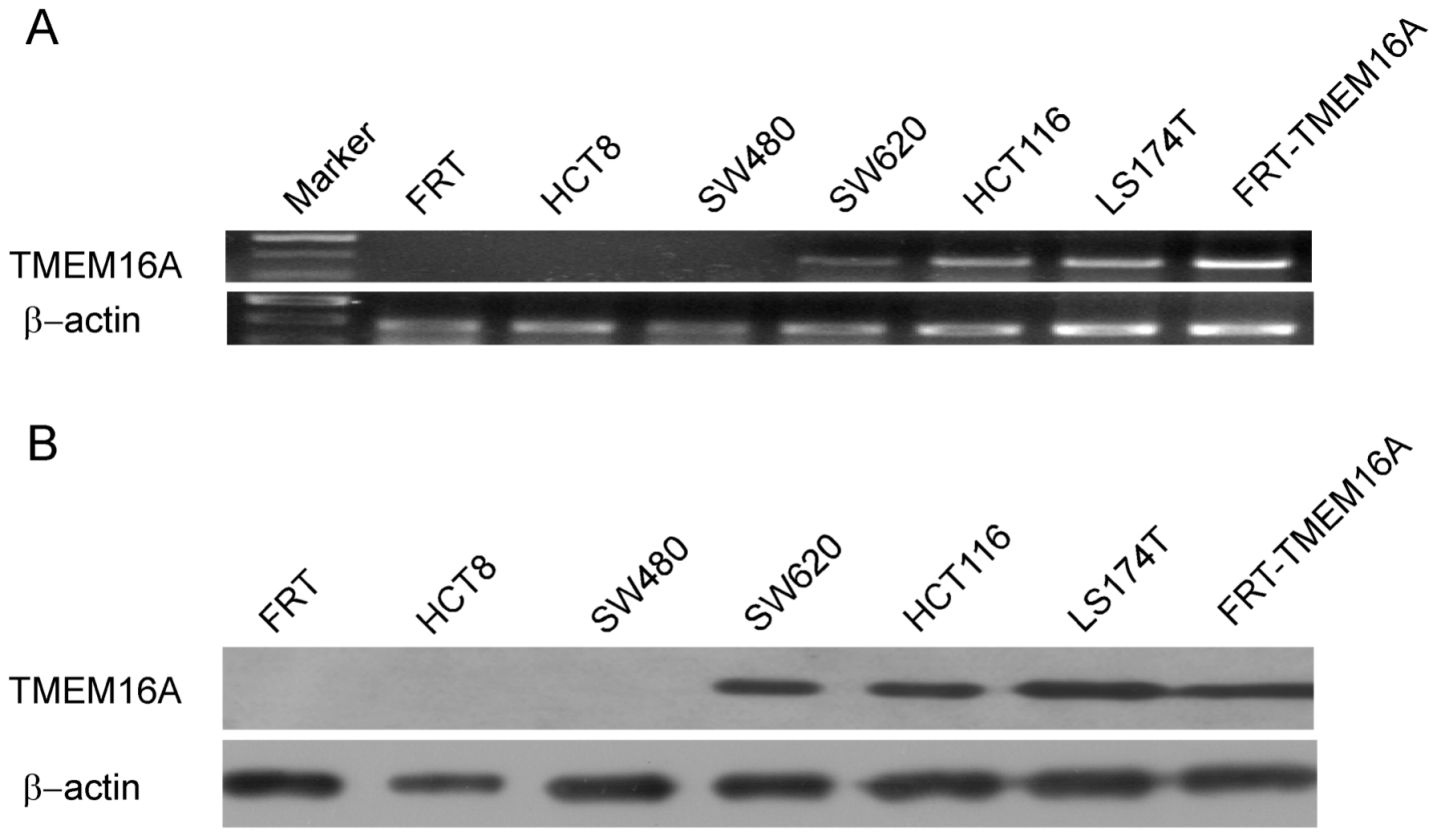
Expression of TMEM16A in CRC cell lines, HCT8, SW480, SW620, HCT116 and LS174T cells. A, RT-PCR reveals TMEM16A mRNA expression in SW620, HCT116 and LS174T cells, but not in HCT8 and SW480 cells. B, TMEM16A protein expression in HCT8 and SW480, SW620, HCT116 and LS174T cells was detected by Western blotting. Null FRT cells acts as negative control. FRT cells transfected with human TMEM16A serve as positive control.

**Figure 2:**
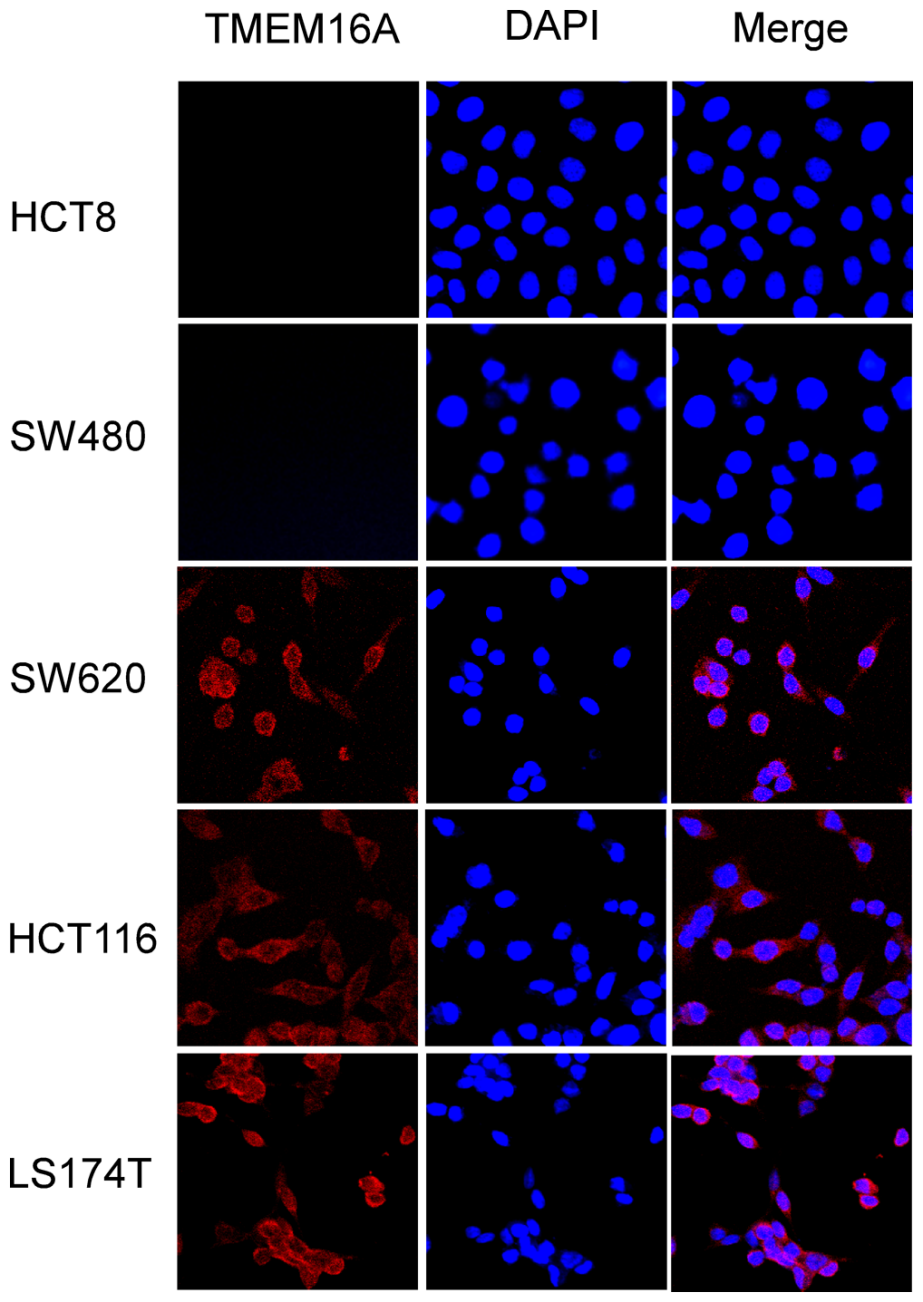
Immunofluorescence labeling of TMEM16A in CRC cell lines. Cells were grown on coverslips and stained with anti-TMEM16A antibodies. Left column, anti-TMEM16A immunofluorescence (Cy3, red). Middle column, DAPI staining to visualize nuclei. Right column, merged images are composites of anti-TMEM16A immunofluorescence and DAPI staining (200×).

In this study, we first detected *TMEM16A* mRNA levels by RT-PCR in the colon cancer cell lines HCT8, SW480, SW620, HCT116 and LS174T cells. As shown in Fig. 1A, a 424-bp fragment of human *TMEM16A* was amplified from SW620, HCT116 and LS174T cells and positive-control FRT cells transfected stably with human *TMEM16A*, but not from HCT8, SW480 cells and negative-control null-FRT cells. Immunoblot analysis using anti-TMEM16A polyclonal antibody identified an approximately 110-kDa protein band in SW620, HCT116 and LS174T cells (Fig. 1B). In contrast, TMEM16A protein band was absent in SW480, HCT8 and negative control null-FRT cells. We further examined the expression and subcellular localization of the TMEM16A protein in CRC cells using immunofluorescent staining. Immunofluorescence labeling revealed that TMEM16A was highly expressed on the cellular membrane and plasma of SW620, HCT116 and LS174T cells (S1 Fig.), whereas TMEM16A was hardly observed in HCT8 and SW480 cells (Fig. 2). A cellular, isogenic model (SW480 and SW620 cell lines) of human CRC metastasis transition was selected for subsequent study.

### Functional expression of the TMEM16A channel in CRC cells

**Figure 3:**
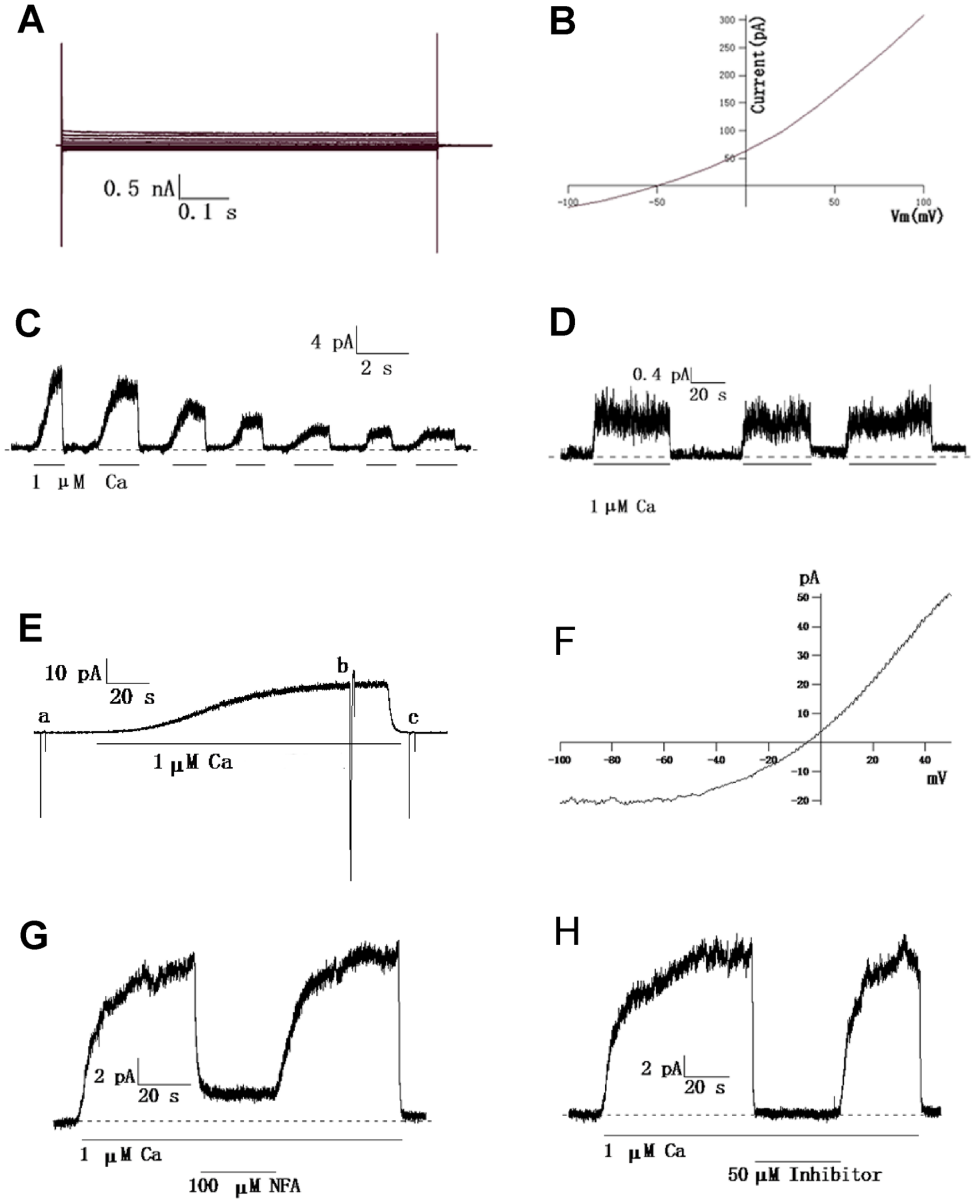
Patch-clamp recording of SW480 and SW620 cells. A, Representative whole-cell currents in SW480 cells were activated by 1 µM Ca. The currents were recorded at a holding potential of 0 mV followed by pulsing voltages between ±100 mV in steps of 20 mV. B, Current-voltage curve from panel A indicating that the currents are not from chloride channels. C, A representative current trace recorded from an inside-out patch of a SW620 cell. A CaCC current was elicited with 1 µM Ca^2+^, as marked. Dashed lines represent zero current. The membrane voltage was held at −50 mV; and upward deflections represent channel opening. D, A stable current trace after a CaCC current from panel C run down. E, A continuous current recording showing activation of CaCCs and applications of voltage ramps in the absence (a and c) or presence (b) of Ca. Voltage ramps: +50 to −100 mV for a duration of 2000 ms. F, Current-voltage curves from panel E showing typical outward rectification characteristics of CaCCs. Note that minimal conductance was observed in the absence of Ca. G and H, CaCCs currents were decreased separately by 100 µM NFA and 50 µM T16Ainh-A01.

To validate the functional expression of the TMEM16A channel in the CRC cell lines, we performed whole-cell patch clamp recordings of SW480 cells and inside-out patch clamp recordings of SW620 cells. Fig. 3A shows a family of currents in response to the voltage steps of stimuli in the presence of 1 µM Ca from a SW480 cell. The current-voltage curve (Fig. 3B) showed that the reversal potential (V_rev_) of this current is approximately −50 mV, but the V_rev_ of the chloride channel was estimated to be approximately 0 mV using the Nernst equation according to the extracellular and intracellular Cl^−^. This result demonstrates that the currents recorded from the SW480 cell are not from chloride channels.

Inside-out patch clamp experiments of SW620 cells manifested that the membrane current was dependent on calcium and the voltage, which are typical characteristics of endogenous CaCCs (Fig. 3C–F). In addition, the detected currents were strongly inhibited by the Cl^−^ channel blocker niflumic acid (NFA) (Fig. 3G) and the TMEM16A -specific inhibitor T16Ainh-A01 (Fig. 3H).

**Figure 4:**
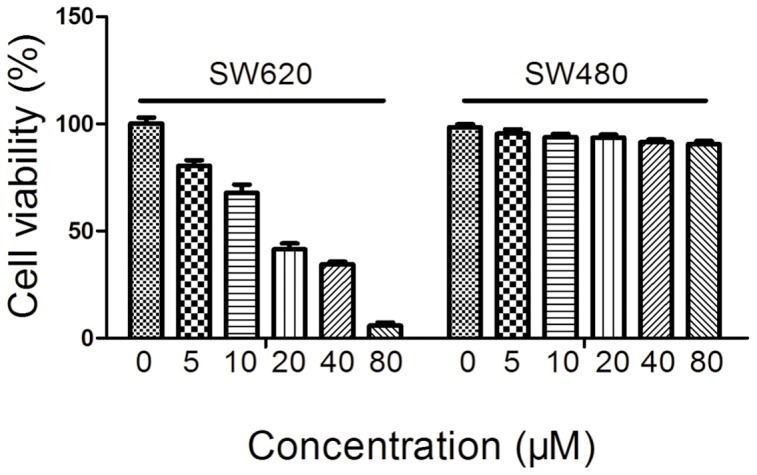
Effect of T16Ainh-A01 on growth of SW480 and SW620 cells. Cells were treated with different concentrations of T16Ainh-A01 for 24 h and cell viability was measured by MTT assay. Data are expressed as Mean ± SD (n = 3).

We also added TEME16A specific inhibitor T16Ainh-A01 to SW620 cells and SW480 cells separately, and observed growth change of these cells after 24 h. The results demonstrated that T16Ainh-A01 could specifically decrease the proliferation of SW620 cells but not SW480 cells (Fig. 4).

### Knockdown of TMEM16A inhibited the proliferation of SW620 cells

**Figure 5:**
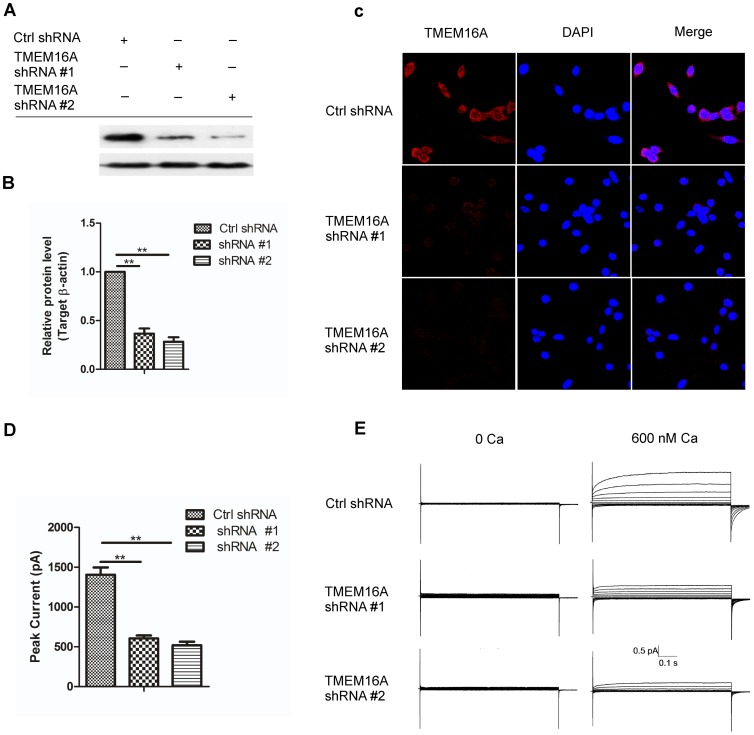
Knockdown of TMEM16A in SW620 cells. A, Western blot analysis of TMEM16A protein expression in SW620 cells that were transfected with shRNA #1 and #2. B, The bar graph summarizes the relative expression level of TMEM16A protein from panel A. Expression of TEMEM16A protein was normalized with the expression level of β-actin (n = 3). C, Representative immunofluence images of SW620 cells are shown after TMEM16A is knockdown by shRNA #1 and shRNA #2, compared with control shRNA (200×). D, The bar graph summarizes the peak currents recorded at 100 mV voltage in SW620 cells after TMEM16A was knocked down, compared with control shRNA. **p<0.01. All data are shown as mean ± SD. E, Whole-cell currents were recorded from SW620 cells transfected with control shRNA, shRNA #1 and #2 at a holding potential of 0 mV followed by pulsing voltages between ±100 mV in steps of 20 mV.

To disclose the functions of TMEM16A in colon cancer cells, we transfected two TMEM16A shRNA sequences (#1 and #2) into SW620 cells to silence the expression of TMEM16A. Western blot results showed that TMEM16A shRNA #1 caused 63.4% reduction in TMEM16A protein expression and TMEM16A shRNA #2 caused 71.7% reduction, as compared with the control shRNA. TMEM16A knockdown led to a significant change in whole-cell calcium-activated chloride currents and immunofluorescene density (Fig. 5).

**Figure 6:**
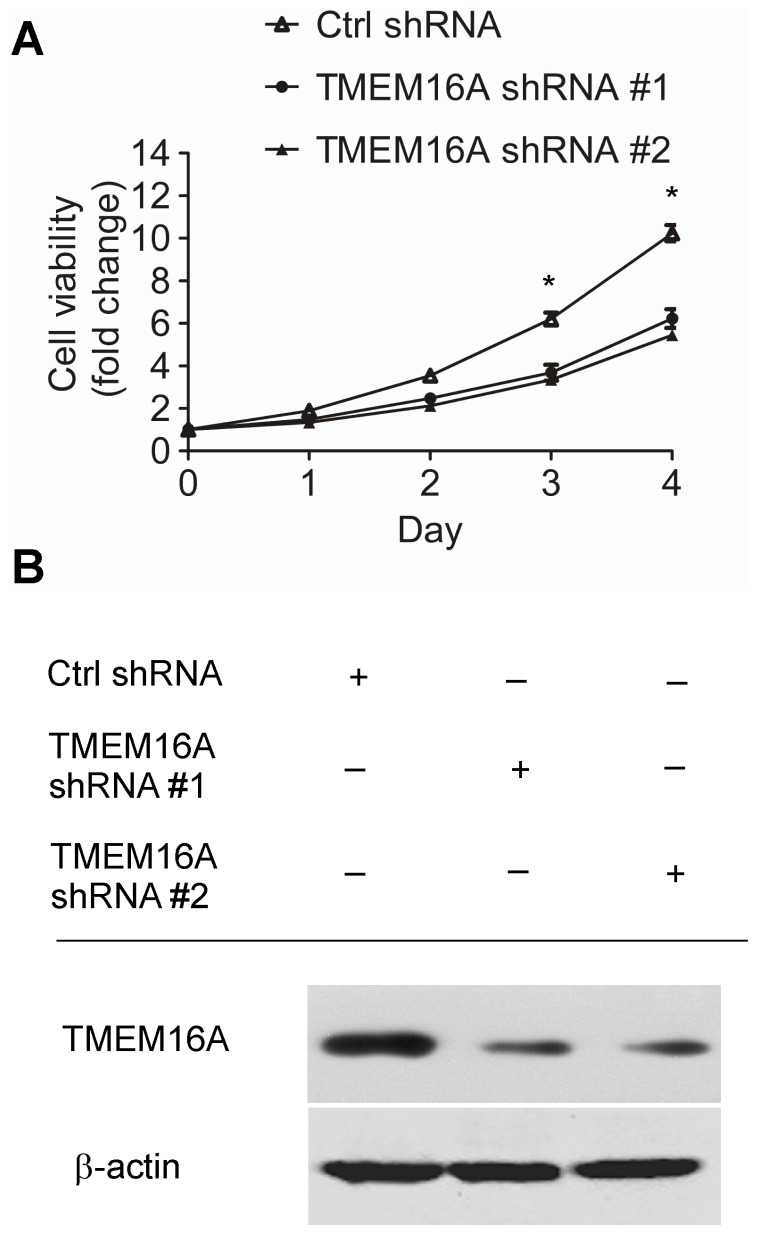
TMEM16A knockdown induced a decrease in proliferation of SW620 cells. A, The growth of SW620 cells was inhibited by TMEM16A shRNA #1 and #2 compared to the control group (Mean ± SD; n = 3; * P<0.05). B, Representative immunoblots confirming knockdown of TMEM16A. Ctrl, control.

To investigate the effects of TMEM16A on the proliferation of SW620 cells, we used two shRNA sequences to interfere the expression of TMEM16A. MTT analysis showed that TMEM16A knockdown greatly suppressed cell growth in a time -dependent manner compared with control shRNA-expressing SW620 cells *in vitro* (Fig. 6).

### Suppression of migration and invasion of SW620 cells by silencing endogenous TMEM16A

**Figure 7:**
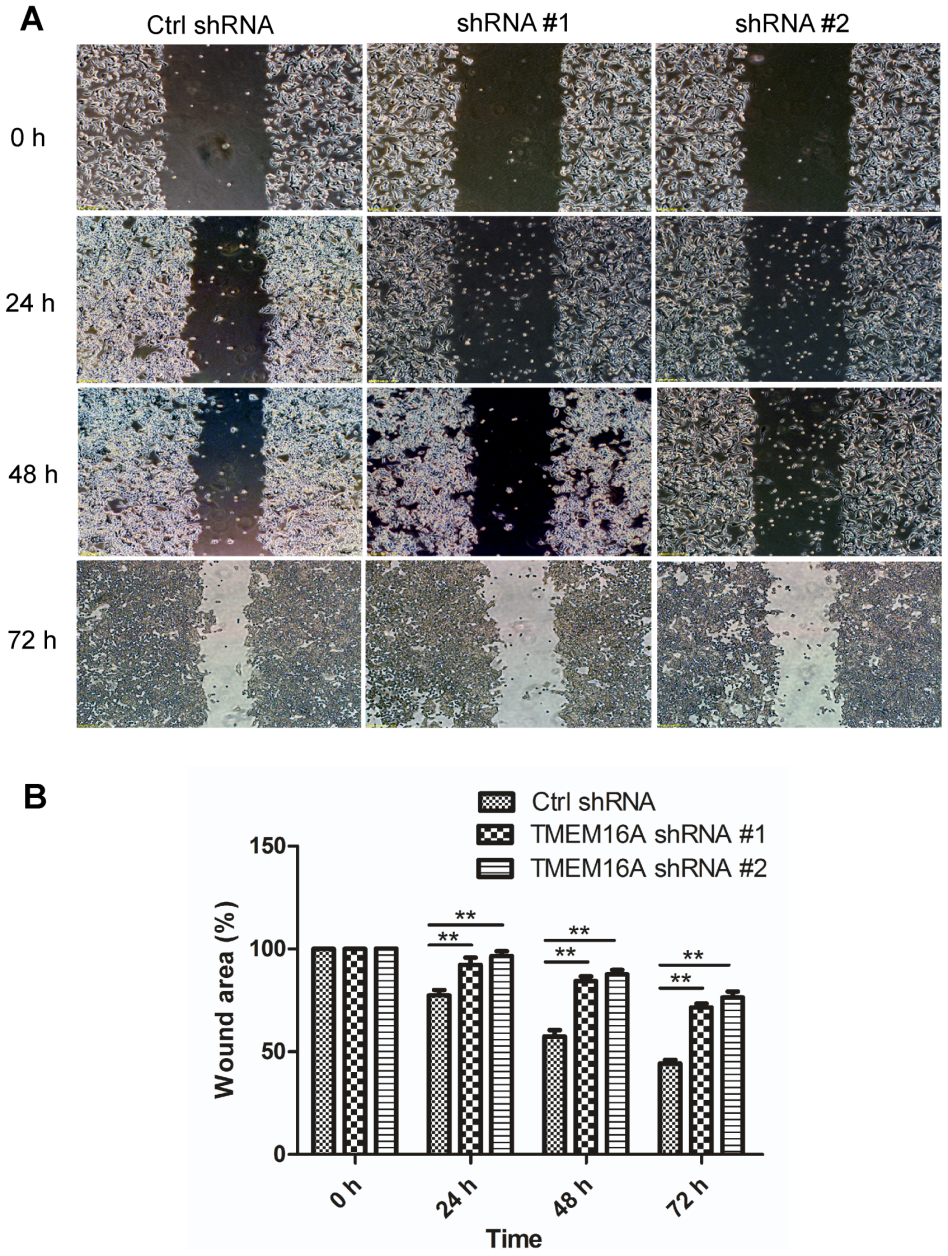
Scratch wound assay of TMEM16A shRNA on SW620 cells. A, Migration of SW620 cells was assessed by a wound-healing assay in the presence of TMEM16A shRNA #1 and shRNA #2, compared with control shRNA. Representative images of wound closure were taken at 0 h, 24 h, 48 h and 72 h after wounding under 100× magnification. B, Bar graphs of panel A are shown. Values are the means ± SD; n = 3; ** P<0.01. Ctrl, control.

To further observe the effects of TMEM16A on cell motility, we performed wound-healing assays after silencing endogenous TMEM16A. Wound-healing images demonstrated that wound closure of TMEM16A shRNA #1 and #2 cells was slower than in the control shRNA-treated cells after scratching the cells (Fig. 7). This result showed that deregulation of TMEM16A delayed wound healing compared with the control.

**Figure 8:**
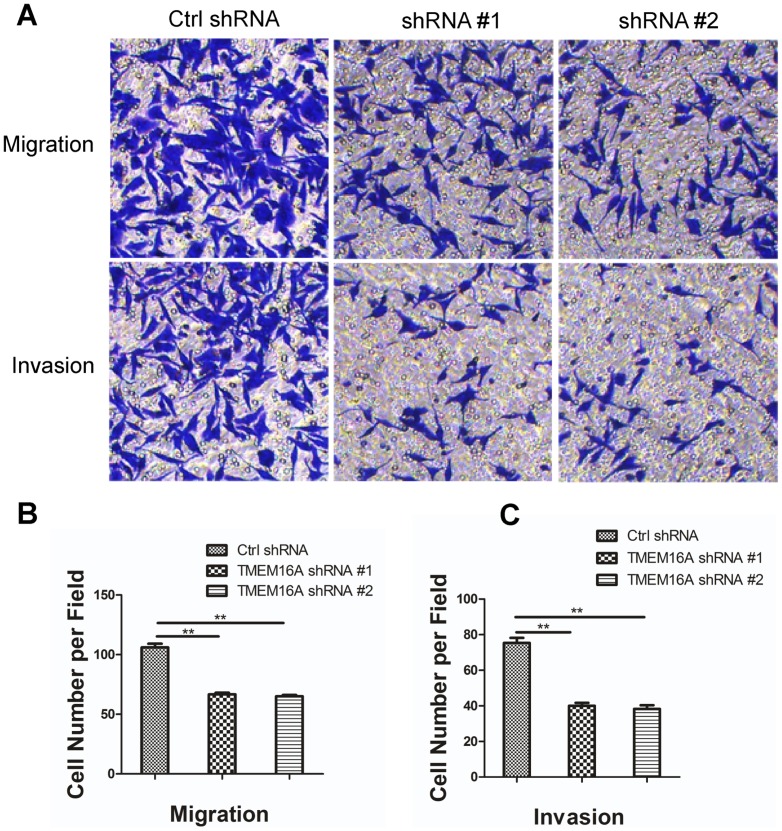
Inhibition of TMEM16A decreased SW620 cell migration and invasion. A, Migration (without Matrigel) and invasion (with Matrigel) of SW620 cells are significantly suppressed by knockdown of TMEM16A compared with the control group through transwell penetration assays. Non-migrated cells were scraped with a cotton swab. Cells that penetrated the transwell filters were stained with Coomassie blue. Representative images are shown. B and C, Bar graphs of panel A are shown. Values are the means ± SD; n = 3; ** P<0.01. Ctrl, control.

We next examined the roles of TMEM16A in the metastasis in SW620 cells. Transwell migration and invasion assays of TMEM16A knockdown cells were carried out. In the transwell migration assay, the numbers of cells transfected with TMEM16A shRNA that penetrated the membrane largely decreased compared with SW620 cells transfected with the negative control. The results also showed that SW620 cells transfected with TMEM16A shRNA #1 and #2 exhibited significant impairment of migratory ability. Similarly, the corresponding role of TMEM16A shRNA #1 and #2 for invasive ability was also observed in a parallel invasive assay (Fig. 8).

### Depletion of TMEM16A inhibited activation of MAPK signaling

**Figure 9:**
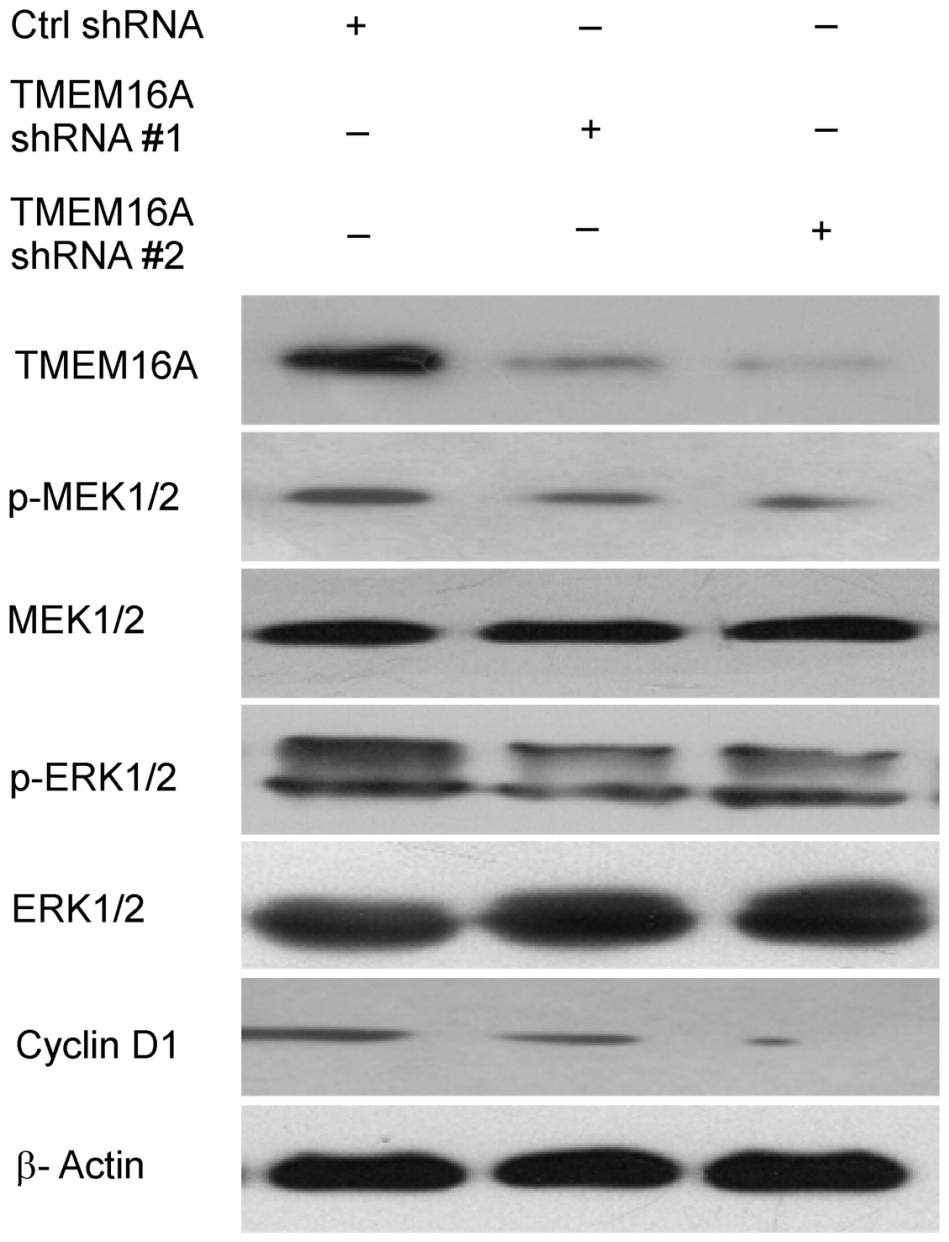
TMEM16A knockdown decreased activation of the MEK-ERK1/2 pathway and expression of cyclin D1. Western blot results show inhibition of TMEM16A led to a decrease in phospho-MEK, phospho-ERK1/2 and cyclin D1. Ctrl, control.

To elucidate the molecular mechanisms by which TMEM16A affects human CRC growth and metastasis, we explored the correlation of TMEM16A with cyclin D1 and MAPK signaling pathway. As shown in Fig. 9, knockdown of TMEM16A significantly decreased the expression of cyclin D1 and phosphorylation of MEK and ERK1/2, whereas it did not affect the total MEK or ERK1/2 expression levels.

### Depletion of TMEM16A caused retardation of cell-cycle procession

**Figure 10:**
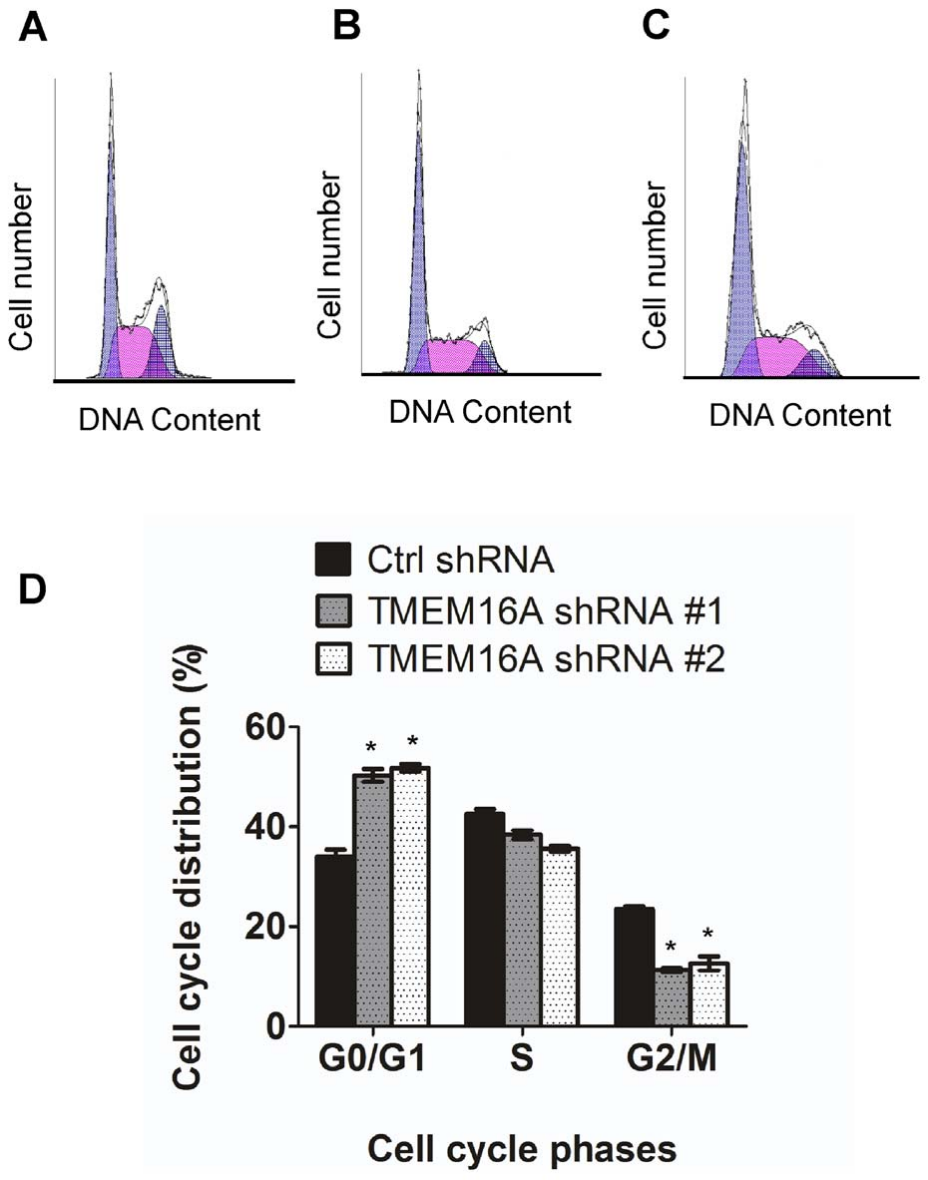
Flow cytometric analysis of the cell cycle distribution in SW620 cells. A, B and C, Flow cytometric analysis of the cell cycle distribution of SW620 cells transfected with control shRNA,TMEM16A shRNA #1 and #2 respectively for 72 h. D, Bar graphs showing the relative percentage of cells in the indicated phases of the cell cycle after knockdown of TMEM16A. Data are expressed as the mean ± SD; n = 3; * P<0.05.

We further measured the cell cycle distribution by flow cytometry to investigate the possible mechanism of TMEM16A in regulating CRC cell proliferation. As Fig. 10 showed, an increase in the cell number at G0/G1 phase and decrease in the cell number at G2/M phase was observed after endogenous TMEM16A was knocked down. The percentage of G1-phase cells in TMEM16A-shRNA #1 cells (50.3%±1.83) and TMEM16A-shRNA #2 cells (51.8±1.13) were significantly higher than in control cells (33.95±2.05) (P<0.01). These results verified that TMEM16A knockdown led to retardation of cell-cycle progression in SW620 cells by arresting cells at Go/G1 phase, which caused the growth inhibition of TMEM16A shRNA cells.

Taken together, our results demonstrate that depletion of TMEM16A suppressed the activation of MAPK signaling pathway and retarded progression of the cell cycle.

## Discussion

TMEM16A proteins were recently found to form Ca^2+^-activated Cl^−^ channels that were transiently activated by intracellular calcium [15], [16], [26], [35]. These channels play an important role in epithelial Cl^−^ secretion, smooth muscle contraction and olfactory signal transduction [17], [36], [37], [38], [39]. Previous studies showed that TMEM16A is frequently over-expressed in several malignancies, including SCCHN, esophageal cancer and gastrointestinal stromal tumors (GIST) [28], [36], [40]. However, the expression of TMEM16A in CRC remains unclear.

Our finding provides the first evidence that TMEM16A is amplified and highly expressed in SW620, HCT116 and LS174T cells but not in HCT8 and SW480 cells. We found that the TMEM16A protein was approximately 110 kD and located in the cellular membrane and plasma of SW620, HCT116 and LS174T cells, which is consistent with previous reports [39], [41]. Colon cancer cell line SW480 originates from primary adenocarcinoma of the colon from a 50-year-old male patient, and SW620 cells are derived from a metastatic mesenteric lymph node of the same patient. So SW620 cells have high metastasis potential compared to SW480 cells [42]. LS174T and HCT116 have higher metastasis potential than HCT8 cells [43], [44], [45]. These results demonstrate that up-regulation of TMEM16A was associated with increased potentiality of metastasis in the five CRC cell lines. We speculate that alteration of TMEM16A expression was associated with the acquisition of more aggressive properties in CRC cell lines, which induces tumors to become metastatic. Relationship between TMEM16A expression and CRC tumor progression needs to be further investigated in more CRC cell lines and in another clinic study.

Owing to their isogenic feature, SW480 and SW620 cell lines serve as a valuable model for studying the genetic alternations in colon cancer metastatic progression [46]. Therefore, we choose this pair of cell model for subsequent study.

Electrophysiological evidence validated that TMEM16A functions as CaCCs in SW620 cells. First, the current is dependent on the concentration of calcium ion in the cytoplasm and voltage (Fig. 3C–F). Moreover, both the chloride channel blocker NFA and TMEM16A-specific inhibitor T16A_inh_-A01 [47] can effectively suppress the currents recorded from a SW620 cell patch (Fig. 3G, H). Interestingly, we observed that after a cell patch was excised, the CaCC current decreased quickly at first and then became relatively stable (Fig. 3C, D). This phenomenon demonstrated that other proteins, such as calmodulin or calmodulin-dependent kinase, might be involved in the regulation of TMEM16A CaCCs in SW620 cells.

There is evidence that chloride channels contribute to tumorigenesis and tumor progression [48], [49], [50], [51]. TMEM16A CaCCs have recently been reported to promote growth and metastasis in HNSCC, prostate cancer and breast cancer [27], [30], [31]. Consistent with these findings, we found that TMEM16A knockdown resulted in the suppression of proliferation, migration and invasion of SW620 cells. Our data provide evidence that TMEM16A likely contributes to tumorigenesis and metastasis in the high-metastatic-potential CRC. Small molecular inhibitors that can decrease the expression of TMEM16A may serve as novel therapeutic drugs for metastatic CRC.

To date, the mechanism by which TMEM16A affects colon cancer remained relatively unexplored. Numerous studies showed that the ERK pathway not only controls cellular proliferation and survival but also influences tumor cell migration, invasion and progression [52], [53], [54]. Recent studies reported that TMEM16A promotes HNSCC tumorigenesis and cancer progression by activating MAPK signaling pathway [30]. Therefore, we focused on studying the relationship between TMEM16A and MEK-ERK1/2 pathway in CRC. Changes in this pathway were investigated after TMEM16A expression was inhibited using western blotting. Our results demonstrate that knockdown of TMEM16A significantly inhibited the activation of MEK and ERK1/2. On the basis of this result, we further investigated the expression of cyclin D1, a cell-cycle regulatory protein that is associated with pERK1/2 expression. The results showed that expression of cyclin D1 is positively associated with pERK1/2 and TMEM16A expression. In addition, flow cytometry (FCM) assays showed that the cell cycle progression from G1 to S phase was inhibited, which was consistent with deregulation of cyclin D1 expression after TMEM16A was depleted. These results provide a possible explanation for the observed inhibition of cell proliferation, migration and invasion of CRC when TMEM16A was suppressed and indicated that TMEM16A contributes to the growth and metastasis of CRC possibly by regulating the MAPK pathway.

In conclusion, we showed that expression of TMEM16A is related to metastatic potential of CRC cell lines. It promotes tumor progression through cell proliferation, migration and invasion. Knockdown of TMEM16A leads to attenuated activation of MEK and ERK1/2 and dysregulation of cyclin D1. These findings highlight the importance of TMEM16A in CRC progression and provide evidence for TMEM16A as a potential therapeutic target of CRC metastasis.

## Supporting Information

S1 Fig **Location of TMEM16A in SW620, HCT116 and LS174T cells.** Cells were grown on coverslips and stained with anti-TMEM16A antibodies. First column, anti-TMEM16A immunofluorescence (Cy3, red). Second column, DAPI staining to visualize nuclei. Third column, images from bright field. Fourth column, merged images from immunofluorescence labeling and bright field. SW620 and HCT116 (200×), LS174T (400×).
(TIF)
