# Supplementary figures and images for: Inhibition of TMEM16A Expression Suppresses Growth and Invasion in Human Colorectal Cancer Cells
Source: PLoS One. 2014 Dec 26;9(12):e115443. doi: 10.1371/journal.pone.0115443 (PMC4277312; doi:10.1371/journal.pone.0115443)

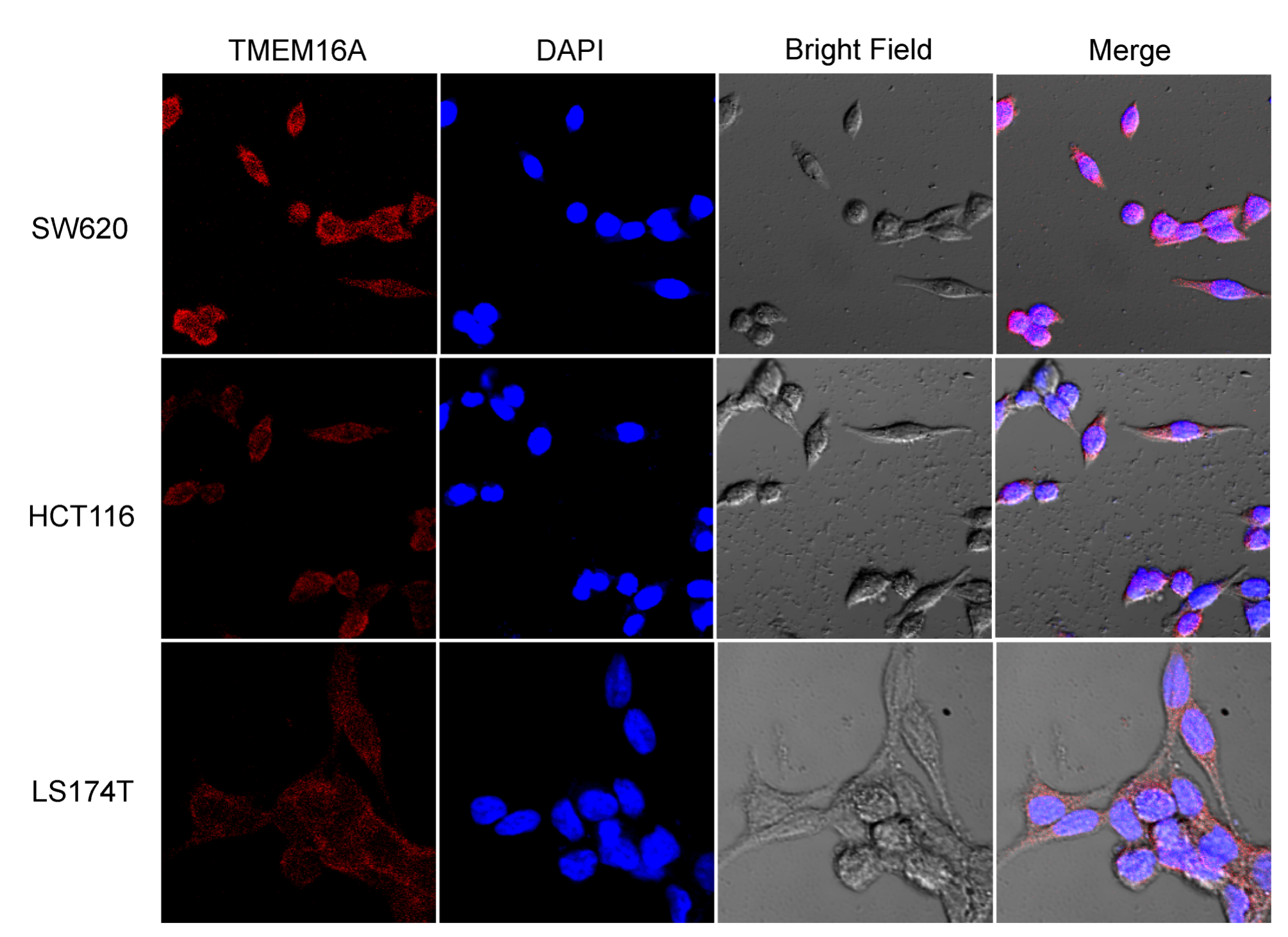

Supplement: S1 Fig — Location of TMEM16A in SW620, HCT116 and LS174T cells. Cells were grown on coverslips and stained with anti-TMEM16A antibodies. First column, anti-TMEM16A immunofluorescence (Cy3, red). Second column, DAPI staining to visualize nuclei. Third column, images from bright field. Fourth column, merged images from immunofluorescence labeling and bright field. SW620 and HCT116 (200×), LS174T (400×). (TIF) [file pone.0115443.s001.tif]
